# Asymmetric Migration of Human Keratinocytes under Mechanical Stretch and Cocultured Fibroblasts in a Wound Repair Model

**DOI:** 10.1371/journal.pone.0074563

**Published:** 2013-09-23

**Authors:** Dongyuan Lü, Xiaofeng Liu, Yuxin Gao, Bo Huo, Yingyong Kang, Juan Chen, Shujin Sun, Li Chen, Xiangdong Luo, Mian Long

**Affiliations:** 1 Key Laboratory of Microgravity (National Microgravity Laboratory), Institute of Mechanics, Chinese Academy of Sciences, Beijing, China; 2 Center of Biomechanics and Bioengineering, Institute of Mechanics, Chinese Academy of Sciences, Beijing, China; 3 Burn Research Institute, Southwest Hospital, Third Military Medical University, Chongqing, China; University of Cambridge, United Kingdom

## Abstract

Keratinocyte migration during re-epithelization is crucial in wound healing under biochemical and biomechanical microenvironment. However, little is known about the underlying mechanisms whereby mechanical tension and cocultured fibroblasts or keratinocytes modulate the migration of keratinocytes or fibroblasts. Here we applied a tensile device together with a modified transwell assay to determine the lateral and transmembrane migration dynamics of human HaCaT keratinocytes or HF fibroblasts. A novel pattern of asymmetric migration was observed for keratinocytes when they were cocultured with non-contact fibroblasts, *i.e.*, the accumulative distance of HaCaT cells was significantly higher when moving away from HF cells or migrating from down to up cross the membrane than that when moving close to HF cells or when migrating from up to down, whereas HF migration was symmetric. This asymmetric migration was mainly regulated by EGF derived from fibroblasts, but not transforming growth factor α or β1 production. Mechanical stretch subjected to fibroblasts fostered keratinocyte asymmetric migration by increasing EGF secretion, while no role of mechanical stretch was found for EGF secretion by keratinocytes. These results provided a new insight into understanding the regulating mechanisms of two- or three-dimensional migration of keratinocytes or fibroblasts along or across dermis and epidermis under biomechanical microenvironment.

## Introduction

Cutaneous wound healing in mammalians, as an intricate and highly-coordinated process, represents a series of sequential chemical and physical events [1]. The classic model of wound healing involves three major phases, including hemostasis and inflammation, formation granulation tissue and re-epithelialization, and matrix formation and remodeling [2], all of which are histologically and functionally different and require interactive, carefully-orchestrated communication of numerous cell types in distinct tissue compartments. Keratinocytes are known to affect greatly the repairing process during these phases, especially in re-epithelialization through their proliferation and migration [3], [4]. For example, re-epithelization stemming from keratinocyte proliferation is a crucial step during wound healing to cover the denuded dermal surface. And keratinocyte migration into wound tissue is essential prior to cell proliferation in the early phase of a few hours after wounding [5].

It has long been noticed that the dynamics of keratinocyte migration is regulated by the local microenvironment [3], [6]. Biochemical signaling is basic, including the communication with other dermal cells, the composition of extracellular matrix (ECM), and the presence of cytokines and growth factors produced by fibroblasts [3], [7]. For example, bi-directional interactions between keratinocytes and fibroblasts become predominant in the mid- and late-phases of wound healing. While a body of evidences indicates that the cocultured keratinocytes induce fibroblasts to synthesize growth factors, which in turn stimulates keratinocyte proliferation via an autocrine, paracrine or the combined manner [8], it is little known how keratinocyte migration is regulated in the presence of fibroblasts and what the key regulators are for keratinocyte and fibroblast interactions. On one hand, different extracellular matrix (ECM) components have distinct effects on keratinocyte motility and migration velocity in the presence of growth factors [9], [10][Bandyopadhyay, 2006 #13;el-Ghalbzouri, 2002 #14;Nickoloff, 1988 #15]. Specifically, fibroblast-secreted type I collagen in wound tissue is one of major ECM components in regulating keratinocytes migration [2]. On the other hand, epidermal growth factor (EGF) family members, such as EGF or transforming growth factor alpha (TGF-α), bind to the EGF receptor (EGFR) and exert important effects in keratinocyte migration and consequently re-epithelialization [11]–[13]. EGF is secreted by fibroblasts and acts in a paracrine manner on keratinocytes for epidermal regeneration after injury [14], [15] to significantly accelerate re-epithelialization [16] and increase tensile strength in wounds [17]. TGF-α is also secreted by fibroblasts and works in a paracrine manner on keratinocytes [15], [18]–[20]. Despite its seemingly important role in re-epithelialization, the absence of this growth factor does not hinder wound healing [21], [22]. Whether EGF and TGF-α are able to accumulate keratinocytes migration and directionality remains to be confirmed. Additionally, TGF-βs, another mediator family for re-epithelialization, are assumed to stimulate re-epithelialization and granulation tissue formation, but the role of TGF-βs in re-epithelialization is controversial in the literatures [23]–[26].

Mechanical signaling is also important in wound healing since skin contraction is pre-requisite to define the function and configuration of regenerative tissue. For example, keratinocyte and fibroblast interactions enhance the contractile activity in fibroblasts under mechanical tension [27]. Mechanical forces generated during tissue development and remodeling modulate the synthesis of various ECM proteins and accelerate the progress of wound healing [3], [4], [28], [29]. Collagen I-mediated keratinocyte migration involves the binding of matrix proteins to adhesive receptors on cell surface where mechanical tension plays a critical role in regulating the de novo synthesis of collagens. In clinic, although the vacuum-assisted closure (VAC, a technique of topical suction pressure therapy) is an effective, widely-applied procedure to promote the healing of various chronic wounds [30], [31], it is still unknown why the mechanical tension exerted by suction pressure is favorable in the VAC therapy at cellular and molecular levels [32].

Thus, the challenging issues for keratinocyte migration in human skin wounds mostly lie in: Whether does the mechanical stretch regulate the ability and dynamics of keratinocyte migration? How do the paracrine of cocultured fibroblasts affect the keratinocyte migration and what are the underlying molecular mechanisms? Here we developed an in vitro tensile device, which is able to quantify, independently or in combination with a transwell chamber, the lateral or transmembrane migration dynamics of human keratinocytes onto collagen I-coated substrate in the presence of human fibroblasts. EGF-mediated asymmetric migration was determined. These results provided a new insight into the mechanism in regulating keratinocyte migration under static stretch, which is potentially applicable in wound healing.

## Materials and Methods

### Cells and reagents

Human dermal fibroblast cell (HF) CRL2088 and spontaneously immortalized keratinocyte cell line (HaCaT) CRL2309 were obtained from American Type Culture Collection (Manassas, VA, USA). HaCaT were cultured in RPMI 1640 medium containing 10% (v/v) heat-inactivated fetal bovine serum (FBS) and 1% penicillin/streptomycin. Fibroblasts were cultured in Dulbecco’s Modified Eagle’s medium (DMEM) supplemented with 10% (v/v) FBS and 1% penicillin/streptomycin for monoculture tests but in RPMI 1640 complete medium for coculture measurements. HaCaT cells at passages 40–45 and HF cells at passages 10–12 were used. All the cells were routinely grown at 37°C in a 5% CO_2_ atmosphere.

RPMI 1640 medium, DMEM, penicillin/streptomycin, PBS were purchased from Hyclone (South logan, UT, USA). FBS was from Gibco (Grand Island, NY, USA). Collagen I was purchased from Sigma-Aldrich (St. Louis, MO, USA). Neutralizing antibodies (Abs) to EGF, TGF-α and TGF-β1 were purchased from R&D Systems (Minneapolis, MN, USA). MTT kit was purchased from Amresco (Solon, OH, USA).

### Mechanical stretch assay for monitoring lateral migration

**Figure 1:**
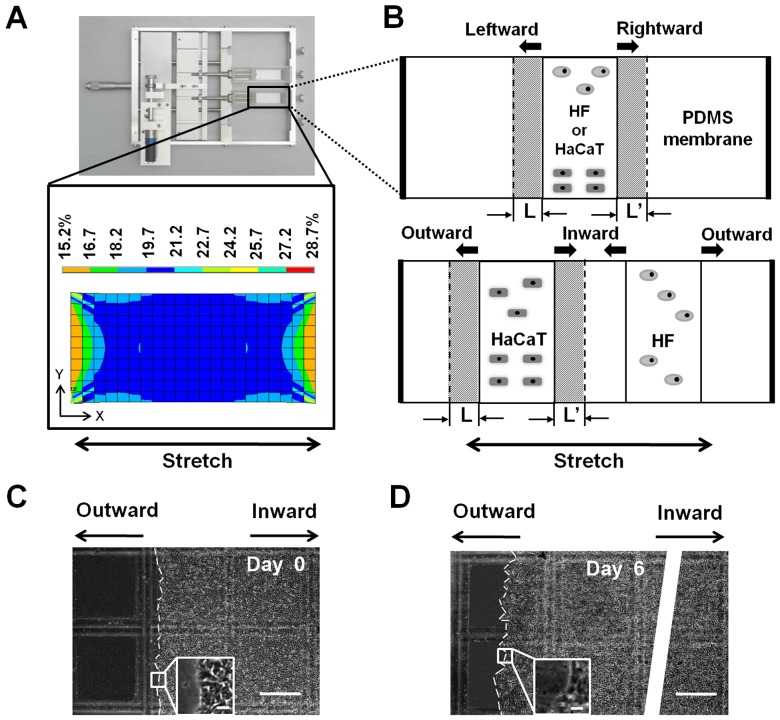
Lateral migration set-up of HaCaT or HF cells cultured on collagen I-coated PDMS membrane in a tensile device. (*A*) An in-house developed tensile device by applying mechanical stretch, *via* the PDMS membrane, to the cells (*upper panel*). Also plotted was the tensile strain profile of PDMS membrane at a pre-set 20% strain along tensile direction (*x*-axis) (*lower panel*). (*B*) Schematic of monocultured (*upper panel*) or cocultured (*lower panel*) HaCaT or HF cells on the stretched membrane where the cells tend to migrate in two directions with a distance *L* or *L’* at a given time point. *Arrows* indicate the migration direction of the cells when they are monocultured (to leftward or rightward) or cocultured (to outward or inward). (*C*, *D*) Typical images of cocultured HaCaT cells at *t* = 0 (*C*) and 6 day (*D*) with the leading edge indicated as *white dashed lines* (*Bar* = 500 µm) with the *inserts* illustrating the magnified images (*Bar* = 100 µm). The *white strip* in (*D*) was used to disconnect the oversized image.

A tensile device was developed for stretching the cells adhering to an elastic membrane in uni-axial manner (Fig. 1). The device is mainly composed of an elastic membrane, a holder box, and a motor, which allows to apply 0–30% strain of mechanical stretch on the cells seeded on the membrane and to take the images of live cells on an inverted microscope. The elastic membrane was made by poly-dimethylsiloxane (PDMS) gel (Sylgard 184, Dow Corning, Midland, MI, USA). Briefly, two components of a “base” and a “curing agent” mixed in a 10:1 ratio (v/v), and the mixture was poured uniformly on the top of a mask suitable for tensile device (width × length  =  20×42 mm in original size). After additional degassing for 12 h, the membrane was cured at 65°C for 3 h and at room temperature (∼25°C) for 12 h. The resulted membrane with a thickness of 3 mm was then coated by collagen I in 0.15 mg/ml at 37°C for 2 h or treated by oxygenized plasma as a control (Fig. 1*A*).

HaCaT and/or HF cells grown up in a flask were harvested using 0.05% trypsin and 0.02% EDTA in phosphate-buffered saline (PBS, Hyclone) when they are approximately 80% confluent, and transferred onto the PDMS membrane mounted on the tensile device. HaCaT and/or HF cells were seeded at respective densities of 5×10^5^ cells/cm^2^ and 1×10^5^ cells/cm^2^. To mimic the distributions of the two types of the cells in wound repair, HF cells were put only on one side of HaCaT cells. In some cases, HaCaT and/or HF cells were first exposed to 10 µg/ml of mitomycin C for 2 h for inhibiting cells proliferation. After being washed three times, collected cells were segregated into two aliquots: one was used for MTT test to examine the inhibition efficiency and the other was employed for stretched-induced migration measurement. For monoculture test, either type of cell was seeded at the center zone of the membrane with an initial width about 8 mm (*upper panel* in Fig. 1*B*). For coculture test, two nontoxic stainless steel frames with same width of ∼8 mm were used to separate the two types of cells symmetrically with a distance of 11.4 mm (*lower panel* in Fig. 1*B*). The membrane was then mounted to the tensile device kept at 37°C in an incubator and experienced a static stretch of 20% strain for up to 6 days.

Cell migration was monitored using a CCD-camera (Olympus, Shinjuku, Tokyo, Japan) in a time interval of ∼24 h. Images were digitalized using Image Pro Plus 6.0 software (Media Cybernetics, Rockville, MD, USA) and the migration distance was measured along the long-axis of PDMS membrane (Figs. 1*C and 1D*). Cell migration distance was estimated as the global displacement of cell monolayer. The capacity of cell migration was measured as normalized fraction, which is defined as follows: For each cell type, the *instantaneous* migration distance at *i*th day was defined as the monolayer width at day *i* subtracted by that at day (*i*-1), which was normalized by the width at day (*i*-1). The *accumulative* migration distance up to day *N* was defined as the sum of the *instantaneous* distances for the period of *t* = 1,,,*i*,,,*N*, which was normalized by the original width of cell monolayer at day 0. Same definition was applied for cocultured cells when the two types of cells migrate separately along the inward (between two cell zones) or outward (away from two cell zones) direction, as well as for monocultured cells when the either type of cells move along the leftward or rightward direction (Movie S1 for leftward and Movie S2 for rightward).

### Modified transwell assay for monitoring transmembrane migration

A conventional transwell assay was modified to study the directed transmigration of HaCaT or HF cells in the presence or absence of HF or HaCaT cells. Briefly, a Boyden chamber insert with a 6.5-mm diameter, l0-µm thickness, porous (8.0-µm) polystyrene membrane (Transwell®, Costar, Tewksbury, MA, USA) was applied in two different ways, namely, *up-to-down* and *down-to-up* transmigrations. For the former, HaCaT or HF cells were first seeded onto the apical surface of the membrane and those cells penetrating the membrane and finally presenting onto the basolateral surface pre-coated by collagen I (0.15 mg/ml) was counted. For the latter, the cells were first seeded onto the basolateral surface of the membrane overnight and then cultured for 48 h after flipping over the insert. The cells finally presenting onto the apical surface pre-coated by collagen I was counted. Note that pre-coating collagen I on the opposite side was designed to induce the transmembrane migration of HaCaT or HF cells. In the coculture tests, HF or HaCaT cells were pre-seeded on the bottom of the well or the PDMS membrane overnight prior to put the transwell insert in.

HaCaT or HF cells were cultured in RPMI 1640 supplemented with 10% FBS and 1% P/S and routinely passaged in a 1:1 ratio for 12–18 h with cycle synchronization by serum-starvation experiments for overnight. 5×10^5^ cells/ml in a volume of 300 µl was added into the designated location of transwell insert or 3×10^4^ cells/cm^2^ cells were placed on the bottom of the well or the PDMS membrane. In parallel, HF cells were seeded on the bottom of the well or the PDMS membrane at 6×10^3^ cells/cm^2^ or 1×10^5^ cells/ml in a volume of 300 µl was added into the designated location of transwell insert. After 24, 36 or 48 h transmigration at 37°C, the filter was fixed with 4% freshly diluted paraformaldehyde. Those non-migrated but attached cells on seeding side were removed with a cotton swab and the remaining migrated cells on opposite side were stained with 0.1% crystal violet for 10 min and washed thrice with water. In each test, eight to twelve images per insert were taken at 40× for the transmigrated cells stained by crystal violet. The number of the cells was counted frame by frame using self-programmed codes and then averaged to obtain the cell number per frame (0.15 mm^2^). The capacity of cell migration was measured as the normalized fraction, which is defined as the ratio of transmigrated cells at a given time point was normalized by that at *t* = 24 h.

### Integration of modified transwell and mechanically-stretched migration

To mimic the three-dimensional (3D) transmigration of keratinocytes through the “keratinocyte-fibroblast barrier” in mechanical microenvironment [33], we further developed an in vitro model by integrating the modified transwell assay with the mechanical stretch assay. The basolateral or apical surface of transwell insert was pre-coated by collagen I to initiate the *up-to-down* or *down-to-up* transmigration when HaCaT or HF cells were seeded on the apical or basolateral side. The insert was then mounted onto the holder box of the tensile device in the presence of HF or HaCaT cells alone or in the presence of both HF or HaCaT cells and mechanical stretch. The gap distance between the transwell membrane and the PDMS membrane is 1.0 mm. In some cases, the Abs to EGF, TGF-α or TGF-β1 was added separately in the medium.

### ELISA measurements for secreted cytokines

To give conclusive information on which type of cells (keratinocytes or fibroblasts) is responsible for the secretion of regulatory growth factors and on what the role of the mechanical stretch plays in the secretion of growth factors, we used a conventional ELISA assay to determine the content of EGF in the culture supernatant in different cases. For the detection of EGF secretion, both two- (2D; lateral migration) and three-dimensional (3D; transmigration) assays were used. In 2D assay, the conditional medium was collected daily in six cases for 5 days: HaCaT M/N (monoculture and non-stretch), HaCaT M/S (monoculture and stretch); HF M/N, HF M/S; HF-HaCaT C/N (coculture and non-stretch), and HF-HaCaT C/S (coculture and stretch). In 3D assay, one type of cell was placed on the bottom of 24-well plate and another type of cells was seeded onto the basolateral side of the transwell. The medium was then collected accumulatively in five cases for 24, 36, 48 h: HaCaT M/N; HF M/N, HF M/S; HF-HaCaT C/N, and HF-HaCaT C/S.

Enzyme linked immunosorbent assay (ELISA) for EGF secretion was carried out using commercial kits from R&D Systems (Minneapolis, MN, USA) upon the manufacturer’s instructions. Briefly, samples and standards (with a dilution series in calibrator diluent) were added to a 96-well microplate and incubated for 2 h at room temperature. After thorough washing, the relevant antibodies were added and incubated for 1 h. Substrate solution containing 1:1 mixture of Color Reagent A (H_2_O_2_) and Color Reagent B (Tetramethylbenzidine) were then added and incubated for 20 min in the dark. The reaction was stopped by adding 2N H_2_SO_4_ and the optical density was determined using Bio-rad iMark template reader at a wavelength of 450 nm with a reference filter at 570 nm.

### Neutralization of growth factors

To test the regulating mechanism of growth factors on the directed migration of HaCaT cells, neutralizing antibodies to human EGF, TGF-α or TGF-β1 were respectively added at a final concentration of 1 µg/ml, upon the manufacturers’ protocols, to provide a minimum of 50% inhibition of cytokine activity. In mechanical stretch assay, anti-EGF Abs was added into the holder box for 6 days. In modified transwell or integrated assay, anti-EGF, TGF-α or TGF-β1 Abs was added into the lower compartment of transwell filter for 48 h. All measurements were done at triplet at 37°C and 5% CO_2_ in a humidified incubator.

### Data analysis

All data were collected from at least triplet measurements and presented as mean ± standard deviation (SD). Analysis of variance (ANOVA) was used to compare the differences among various groups, and Student *t*-test was employed to compare the difference between two groups. *P* value indicates the level of statistical significance of differences in the normalized distance or fraction. Tests that produce *P*<0.01 in mechanical stretch assay or *P*<0.01 in modified transwell or integrated assay were considered to be significant.

## Results

### Lateral migration of HaCaT cells is asymmetric in the presence of HF cells

**Figure 2:**
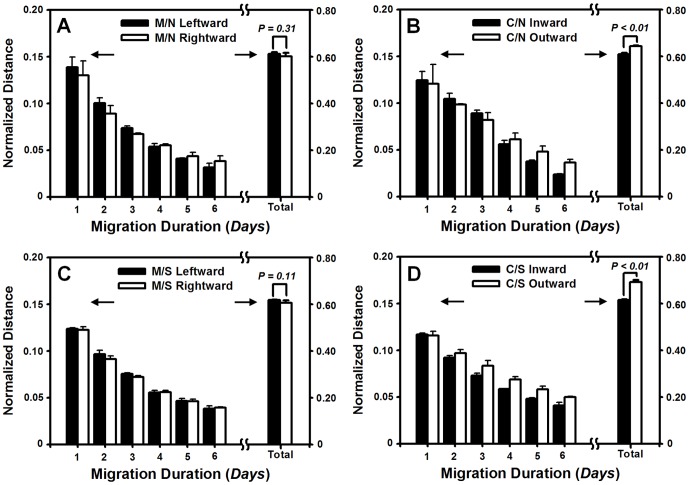
Lateral migration of HaCaT cells on collagen I-coated PDMS membrane in the absence or presence of HF cells and mechanical stretch. (*A, B*) Time course of normalized *instantaneous* or *accumulative* distance of monocultured (M/N) (*A*) or cocultured (C/N) (*B*) HaCaT cells in the absence of mechanical stretch. (*C*, *D*) Time course of normalized *instantaneous* or *accumulative* distance of monocultured (M/S) (*C*) or cocultured (C/S) (*D*) HaCaT cells in the presence of mechanical stretch. Data were collected from at least triplets and presented as the mean±standard deviation (SD) of migration distance and then normalized by the initial width at *t* = 0 day. *P* value indicates the level of statistical significance of difference in normalized distances between leftward and rightward migration for M/N and M/S or inward and outward migration for C/N and C/S. *Arrows* indicate the different scales of the double y-axes for normalized *instantaneous* and *accumulative* distances.

We first tested the lateral migration of HaCaT cells on the collagen I-coated PDMS membrane in the absence or presence of HF cells. Typical images of cocultured HaCaT cells migrating onto the membrane were presented at *t* = 0 (Fig. 1*C*) and 6 day (Fig. 1*D*), respectively. While HaCaT or HF cells were placed separately in the central zone or seeded simultaneously with pre-set separating distance, the cells were allowed to migrate bi-directionally along the long axis of the membrane. It was indicated that, for HaCaT cells alone (M/N), the normalized *instantaneous* distance was comparable along left or right direction up to six days, resulting in the indifferent normalized *accumulative* distance (0.61±0.01 *vs*. 0.60±0.02, *P* = 0.31) (Fig. 2*A*). When both HaCaT and HF cells were placed simultaneously (C/N), the time course of inward (the direction of HaCaT cells migration is close to cocultured HF) or outward (the direction of HaCaT cells migration is away to cocultured HF) migration showed that the outward *instantaneous* distance is slightly higher than the inward distance at the late phase (*t* = 4–6 day) though no significant differences were found in between at the early phase (*t* = 1–3 day). This observation was confirmed by the *accumulative* distance with the higher outward value than that for inward one (0.64±0.00 *vs*. 0.61±0.01, *P*<0.01) (Fig. 2*B*), implying that the outward movement be favorable and the lateral migration be asymmetric in the presence of HF cells.

### Asymmetry of lateral migration of HaCaT cells was mechanically enhanced

Next, we used a tensile device to test the impact of mechanical stretch on lateral migration of HaCaT cells (Fig. 1*A*). A 20% strain of static stretch was applied by pulling the PDMS membrane mounted on the device (*upper panel*) and the cells anchored on the membrane were then subjected to mechanical stretch. Numerical simulations using finite element analysis indicated that the membrane strain distributed uniformly across the membrane except of those regions near two ends, ensuring that a uniform stretch is achieved at least within the central zone of 20×34 mm (*lower panel*). HaCaT and/or HF cells were then placed within this zone to test the cell migration under mechanical stretch.

Migration dynamics of monocultured or cocultured HaCaT cells was compared in the presence of mechanical stretch. For HaCaT cells alone (M/S), no difference in *instantaneous* or *accumulative* distance was found for the cells moving to the left or right (0.62±0.00 *vs*. 0.61±0.01, *P* = 0.11) (Fig. 2*C*). In the case that both HaCaT and HF cells were stretched (C/S), the *accumulative* distance was higher for outward migration than that for inward migration (0.69±0.01 *vs*. 0.62±0.00, *P*<0.01) (Fig. 2*D*), imparting the confidence that the lateral migration of HaCaT cells is asymmetric. Moreover, the outward migration distance was enhanced (0.69±0.01 *vs*. 0.64±0.00, *P*<0.01) but the inward migration was comparable (0.62±0.00 *vs*. 0.61±0.01, *P* = 0.22) in the presence of stretch, as compare to those values in the absence of stretch (*cf*. Figs. 2*A, B*), suggesting that the asymmetric migration of cocultured HaCaT cells be fostered by exerting mechanical stretch.

It is well known that ECM components are crucial to the cell migration [34]. Thus, we tested if the ECM absence on the membrane reduces or eliminates the asymmetric migration. It was found that, on the oxygenized but not collagen I pre-coated PDMS membrane, the *instantaneous* distance fluctuated with time and the *accumulative* one was indifferent along left or right direction for monocultured HaCaT cells in the absence (Fig. *S*1*A*) or presence (Fig. *S*1*C*) of mechanical stretch. Similar indifference between outward and inward migration was also observed for cocultured HaCaT cells in the absence of stretch (Fig. *S*1*B*). This symmetric migration on oxygenized membrane was distinct from the above asymmetric migration on collagen-I coated membrane (*cf*. Fig. 2*B*), suggesting that collagen I is vital to the asymmetric migration of the cells. Interestingly, the asymmetric migration of cocultured HaCaT cells was presented on oxygenized membrane by applying a mechanical stretch, as seen in the different accumulative distance along outward or inward direction (0.66±0.03 *vs*. 0.58±0.01, *P* = 0.02) (Fig. *S*1*D*), supporting that mechanical stretch is also critical to represent the asymmetric migration of HaCaT cells.

Another possible mechanism for such an asymmetric migration may be attributed to the distinct capabilities of cell proliferation along the opposite directions. To exclude this possibility, both HaCaT and HF cells were pre-incubated with a proliferation inhibitor of alkylating agent, mitomycin C, before exerting mechanical stretch. It was indicated that cell migration, even slightly lower than that in the absence of the inhibitor, was still remarkable (Fig. *S*2). Specifically, the outward and inward *accumulative* distances of HaCaT migration were indifferent in the absence of stretch (0.55±0.00 *vs*. 0.54±0.00, *P* = 0.16) (Fig. *S*2*A*), but the migration revealed an asymmetric feature in the presence of stretch (0.58±0.00 *vs*. 0.55±0.01, *P*<0.01) (Fig. *S*2*B*). These results implied that the migration magnitude is dependent on but the asymmetric feature is independent on cell proliferation. It was also evident that the mechanical stretch is required for asymmetric migration of HaCaT cells.

### Lateral migration of HF cells is symmetric

**Figure 3:**
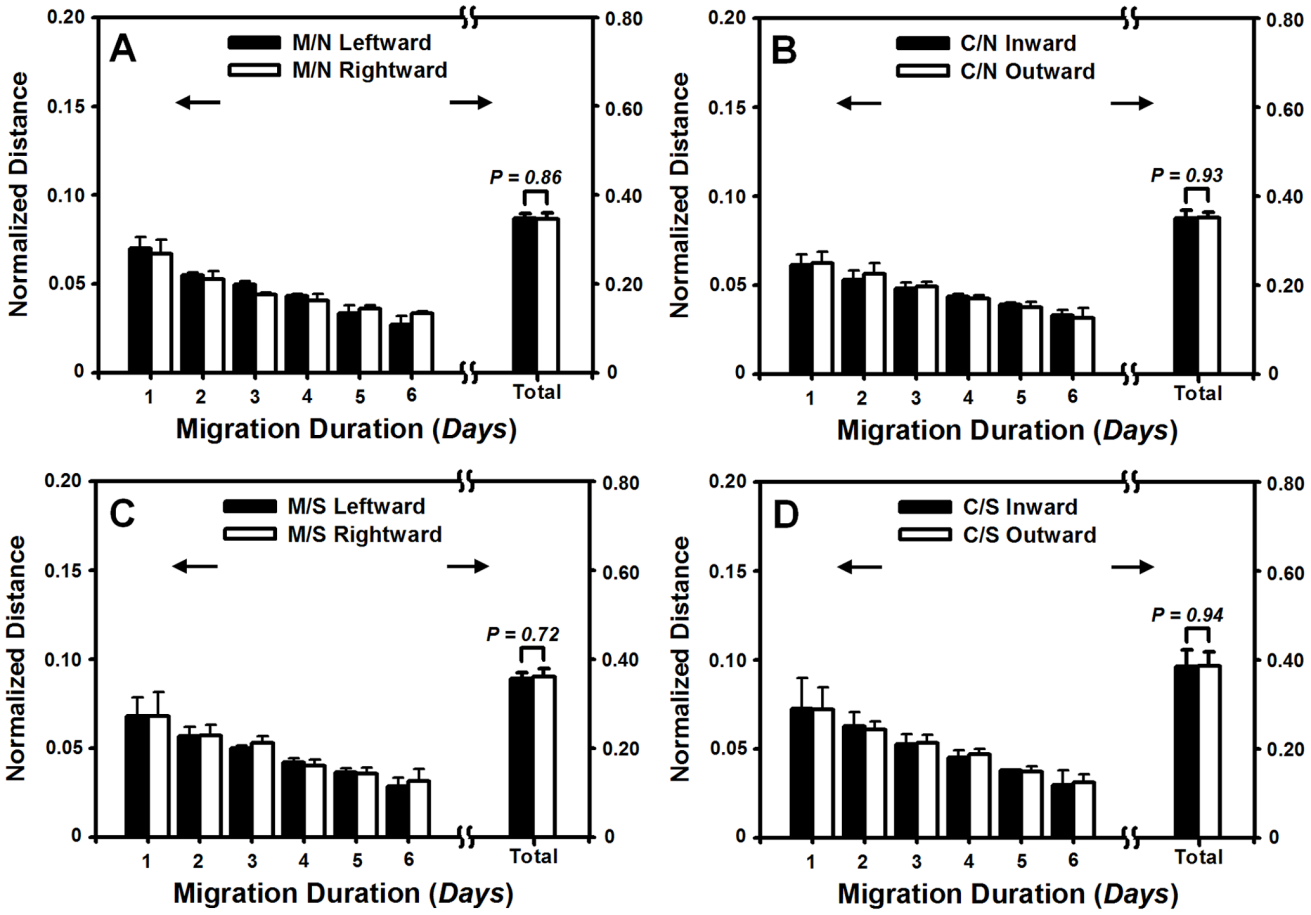
Lateral migration of HF cells on collagen I-coated PDMS membrane in the absence or presence of HaCaT cells and mechanical stretch. (*A, B*) Time course of normalized *instantaneous* or *accumulative* distance of monocultured (M/N) (*A*) or cocultured (C/N) (*B*) HF cells in the absence of mechanical stretch. (*C*, *D*) Time course of normalized *instantaneous* or *accumulative* distance of monocultured (M/S) (*C*) or cocultured (C/S) (*D*) HF cells in the presence of mechanical stretch. Data were collected and presented in the same way as Fig. 2.

We also performed the measurements for lateral migration of HF cells on collagen I-coated PDMS membrane. Our data indicated that the normalized *instantaneous* distance was comparable between leftward and rightward migration for up to six days, resulting in the identical normalized *accumulative* distance (0.35±0.01 *vs*. 0.35±0.01, *P* = 0.86) in M/N (Fig. 3*A*). When both HF and HaCaT cells were placed simultaneously (C/N), the time courses of inward and outward migration were also indifferent and yielded the same *accumulative* distance (0.35±0.02 *vs*. 0.35±0.01, *P* = 0.93) (Fig. 3*B*). Similar measurements were performed by applying mechanical stretch. Again, no significant difference in *accumulative* distance was found for HF cells alone between leftward and rightward migration (M/S) (0.36±0.01 *vs*. 0.36±0.02, *P* = 0.72) (Fig. 3*C*) or HF cells cocultured with HaCaT cells between inward and outward migration (C/S) (0.38±0.04 *vs*. 0.39±0.03, *P* = 0.94) (Fig. 3*D*). These results suggested the lateral migration of HF cells is symmetric.

We also tested if the ECM absence on the membrane to influence HF migration. It was found that, on the oxygenized but not collagen I pre-coated PDMS membrane, the *instantaneous* distance fluctuated with time and the *accumulative* one was indifferent along left or right direction for monocultured HF cells in the absence (Fig. *S*3*A*) or presence (Fig. *S*3*C*) of mechanical stretch. Similar indifference between outward and inward migration was also observed for cocultured HF cells in the absence (Fig. *S*3*B*) or presence (Fig. *S*3*D*) of mechanical stretch. This *instantaneous* distance fluctuated with time on oxygenized membrane was distinct from the above HF migration on collagen-I coated membrane (*cf*. Fig. 3), suggesting that collagen I is important to the migration of the cells.

### Transmembrane migration of HaCaT cells is also asymmetric

**Figure 4:**
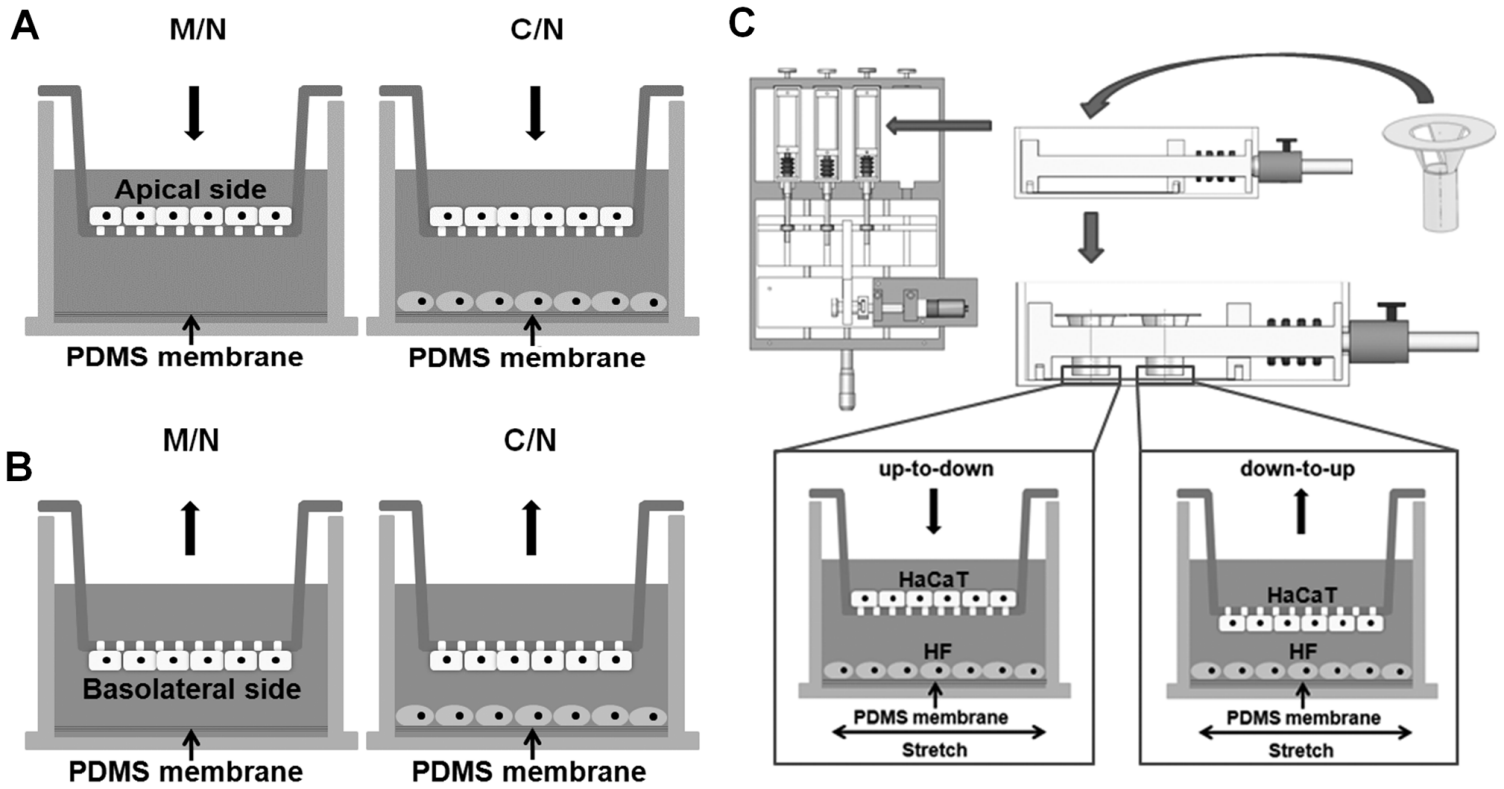
Transmembrane migration set-up of HaCaT or HF cells using a modified transwell assay. Plotted was *up-to-down* (*A*) or *down-to-up* (*B*) transmigration of monoclutured or cocultured HaCaT or HF cells in the absence or presence of mechanical stretch. Integration of the mechanical stretch assay with the modified transwell assay was also illustrated (*C*) and the detailed protocols were referred in Fig. *S*4.

**Figure 5:**
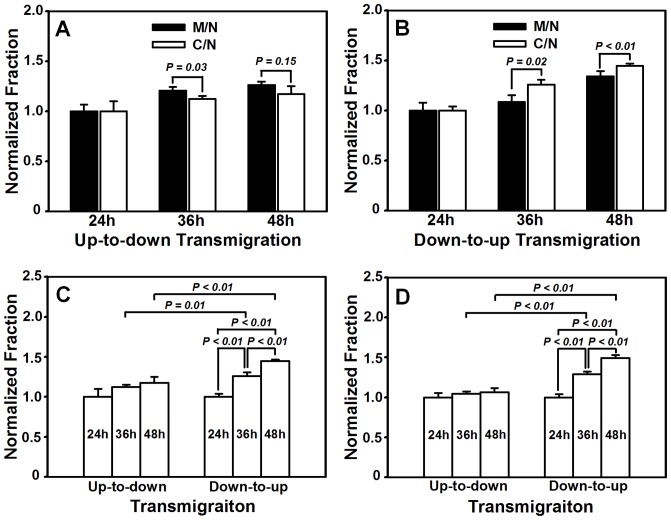
Transmigration of HaCaT cells on collagen I-coated transwell chamber. (*A, B*) *Up-to-down* (*A*) or *down-to-up* (*B*) transmigration of monoclutured or cocultured HaCaT cells in the absence of mechanical stretch. (*C*, *D*) *Up-to-down* or *down-to-up* transmigration of cocultured HaCaT cells in the absence (*C*) or presence (*D*) of mechanical stretch. Data are presented as the fraction of transmigrated HaCaT cells at *t* = 36, 48 h normalized to that at *t* = 24 h in respective cases.

In wound healing, not only lateral migration of keratinocytes and fibroblasts is crucial under skin tension, but the cells also migrate along the direction perpendicular to skin surface and cross over dermis and epidermis. While it is hard to replicate, *via* an *in vitro* assay, such a physiological 3D cell migration across dermis and epidermis tissue, we attempted to test if the asymmetric migration of HaCaT cells is also observed in a 3D-mimicking microenvironment. Here a conventional transwell assay was modified to test the HaCaT cell transmigration in the two manners, that is, *up-to-down* (Fig. 4*A*) and *down-to-up* (Fig. 4*B*) transmembrane migration (Fig. *S*4). Images of transmigrated cells illustrated in Fig. *S*5 were used to count the cells from either side. The number of cells having migrated to the collagen-I pre-coated basolateral or apical surface was then counted at given time point. This value was then normalized by the one at *t* = 24 h to compare the directed transmigration. Data indicated that, in the absence of mechanical stretch, the transmigration of monocultured or cocultured cells increased with time (Figs. 5*A, B*). Moreover, the presence of HF cells on the bottom of non-stretched PDMS membrane reduced the *up-to-down* transmigration of HaCaT cells. The normalized fraction was lower at *t* = 36 h (1.12±0.03 *vs*. 1.21±0.04, *P*<0.05) or comparable at *t* = 48 h (1.17±0.08 *vs*. 1.26±0.04, *P*>0.10) for cocultured cells than that for monocultured cells (Fig. 5*A*). By contrast, the *down-to-up* transmigration of HaCaT cells was enhanced in the presence of HF cells, resulting in the higher values than those in the absence of HF cells at *t* = 36 h (1.26±0.05 *vs*. 1.09±0.07, *P*<0.05) or 48 h (1.45±0.02 *vs*. 1.24±0.05, *P*<0.01) (Fig. 5*B*).

To further test if the transmigration of HaCaT cells is asymmetric, we re-plotted the data of cocultured HaCaT cells shown in Fig. 5*A, B* and compared the fraction of transmigrated HaCaT cells between the two manners. At the same time point, the coculture of HaCaT cells with HF cells yielded the higher fractions in *down-to-up* transmigration than those in *up-to-down* transmigration at *t* = 36 h (1.26±0.05 *vs*. 1.12±0.03, *P*<0.02) or 48 h (1.45±0.02 *vs*. 1.17±0.08, *P*<0.01), suggesting that *down-to-up* migration was in preference to *up-to-down* migration (Fig. 5*C*). By contrast, both *up-to-down* and *down-to-up* migrations for monocultured HaCaT cells were comparable at *t* = 36 h (1.09±0.07 *vs*. 1.21±0.04, *P* ∼ 0.05) or *t* = 48 h (1.24±0.05 *vs*. 1.26±0.04, *P*>0.46). Taken together, these results implied that the HaCaT transmigration is also asymmetric, at least for cocultured cells. These data also indicated that the fraction of transmigrated cells is higher in *down-to-up* than that in *up-to-down* in the presence of HF cells (Fig. 5*C*) whereas no significant difference was found in between in the absence of HF cells (Figs. 5*A, B*).

### Asymmetry of HaCaT transmembrane migration is mechanically modulated

To further test the impact of skin tension on HaCaT transmigration, we integrated the modified transwell assay with the mechanical stretch assay (Figs. 4*C*, *S*4) to mimic the 3D transmigration of keratinocytes under 20% strain of static stretch. It was indicated that, while the fraction of transmigrated cells was comparable within the value of 1.00–1.06 at *t* = 24, 36, or 48 h in *up-to-down* migration (all the values, *P* = 0.24–0.62), it dramatically increased from 1.00 to 1.49 with time in *down-to-up* migration (all the values, *P*<0.01) (Fig. 5*D*). These data suggested that the transmigration dynamics of HaCaT cells between the two manners is distinct in the presence of mechanical stretch.

We also compared the fraction of transmigrated cells between the two manners at the same time point. It was found that the fraction of *down-to-up* transmigration was higher than that for *up-to-down* transmigration at *t* = 36 h (1.29±0.03 *vs*. 1.04±0.03, *P*<0.01) or *t* = 48 h (1.49±0.04 *vs*. 1.06±0.05, *P*<0.01) (Fig. 5*D*), further supporting that the HaCaT transmigration is asymmetric.

### Transmembrane migration of HF cells is symmetric

**Figure 6:**
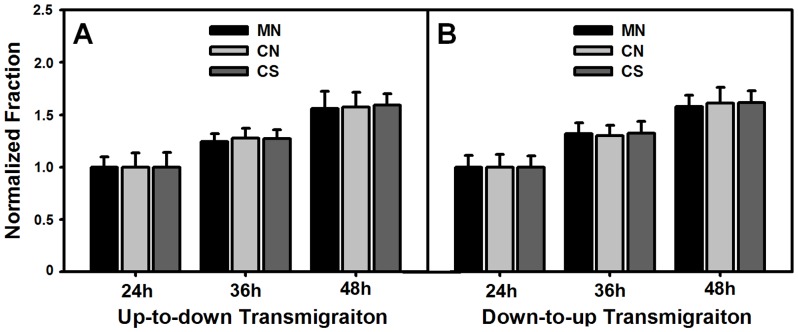
Transmigration of HF cells on collagen I-coated transwell chamber. *Up-to-down* (*A*) or *down-to-up* (*B*) transmigration of monoclutured or cocultured HF cells in the absence or presence of mechanical stretch. Data are presented as the fraction of transmigrated HaCaT cells at *t* = 36, 48 h normalized to that at *t* = 24 h in respective cases.

We also tested transmigration of HF cells, as done for HaCaT cells described above. Again, our data indicated that the transmigration of monocultured or cocultured HF cells increased with time in the absence or presence of mechanical stretch (Figs. 6*A, B*). Normalized fraction of transmigrated cells was comparable at given time points, which yields 1.25–1.27 (*P* = 0.24–0.62) at *t* = 36, or 1.56–1.59 (*P* = 0.80–0.92) at *t* = 48 h in *up-to-down* transmigration (Fig. 6*A*), and 1.30–1.32 (*P* = 0.79–0.96) at *t* = 36 h or 1.58–1.62 (*P* = 0.69–0.96) at *t* = 48 h in *down-to-up* transmigration (Fig. 6*B*). We also compared the fraction of transmigrated HF cells between the two manners. At the same time point, both *up-to-down* and *down-to-up* transmigrations of HF cells yielded the similar values in all the three cases of M/N, C/N, and C/S, that is, 1.25±0.07 and 1.32±0.10 (*P* = 0.38) at *t* = 36 h or 1.56±0.16 and 1.58±0.11 (*P* = 0.90) at *t* = 48 h for monoculture and non-stretch (M/N), 1.28±0.10 and 1.30±0.10 (*P* = 0.81) at *t* = 36 h or 1.57±0.14 and 1.61±0.15 (*P* = 0.78) at *t* = 48 h for coculture and non-stretch (C/N), and 1.27±0.09 and 1.32±0.11 (*P* = 0.56) at *t* = 36 h or 1.59±0.11 and 1.62±0.11 (*P* = 0.81) at *t* = 48 h for coculture and stretch (C/S). Taken together, these results implied that the transmigration of HF cells is symmetric regardless of the existence of HaCaT cells or mechanical stretch or not.

### EGF is mainly secreted by HF cells and up-regulated by mechanical stretch

**Figure 7:**
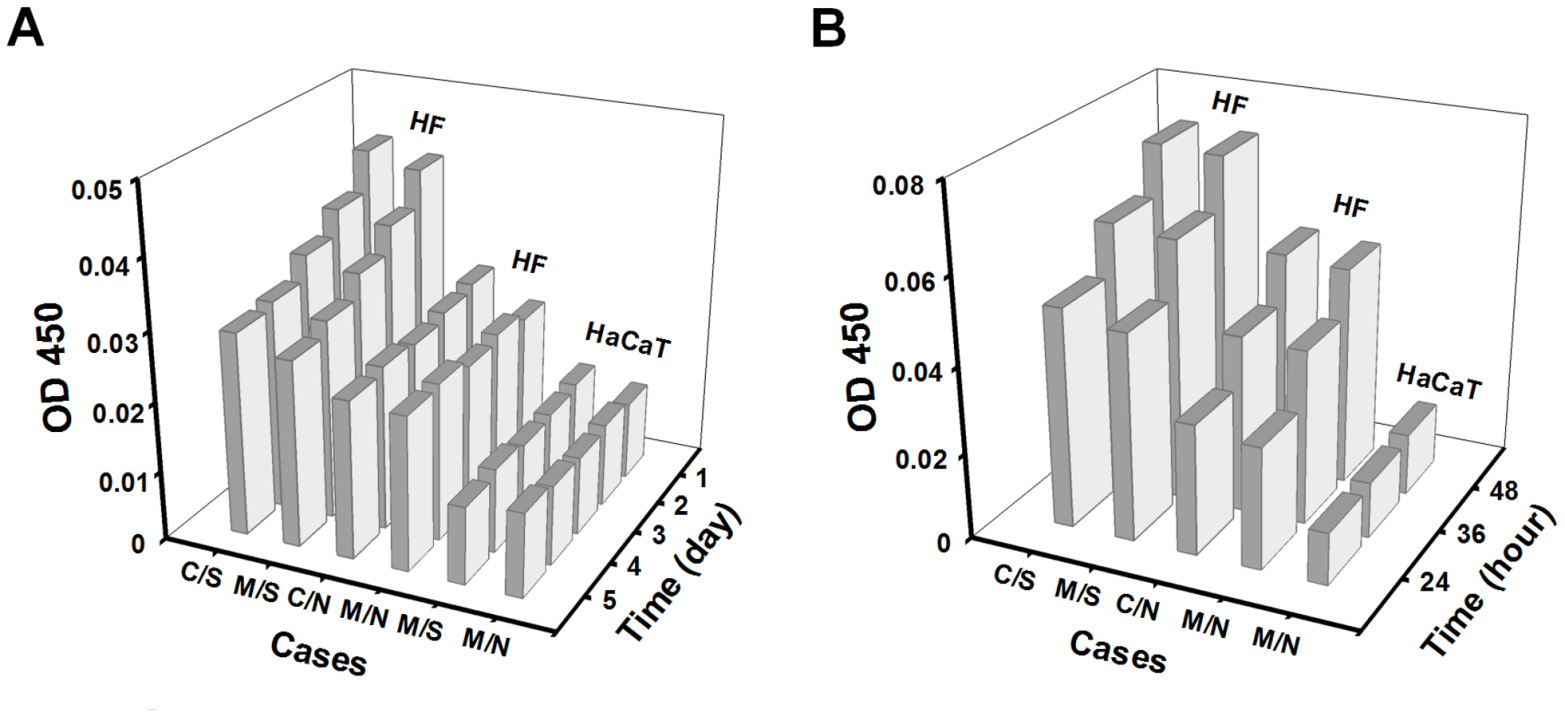
Comparison of content of EGF secretion in the conditional medium using 2D (*A*) or 3D (*B*) assay. Data were collected from at least triplets and presented as the average optical intensity at OD 450

Growth factors secreted by either HaCaT or HF cells are crucial to regulate the re-epithelialization in wound healing. Here we hypothesized that epithelial growth factor or EGF may manipulate the lateral and/or transmembrane migration of the cells. EGF secretion was systematically tested using a conventional ELISA assay for the two types of cells in both 2D and 3D assays. In 2D assay (Fig. 7*A*), for example, the mean EGF content of daily secretion retained the same baseline values at various time points for monocultured HaCaT cells in the absence or presence of mechanical stretch. It yielded significantly higher values for HF cells alone or cocultured with HaCaT cells even though no difference was found for the time course of daily secretion. Moreover, the action of mechanical stretch further enhanced the EGF content for HF cells alone or cocultured with HaCaT cells, and also promoted the daily-decay dependence of EGF secretion at day 1–2 and the equilibrium secretion at day 3–5. No difference was found between each two subgroups in all the three cases (Fig. 7*A*). In 3D assay, the mean EGF content of accumulative secretion also reserved a baseline value at different time points for monocultured HaCaT cells in the absence of mechanical stretch. It increased with time linearly for HF cells alone or cocultured with HaCaT cells and was further enhanced when the mechanical stretch was present. Again, no difference was found between each two subgroups in the latter two cases (Fig. 7*B*). Taken together, EGF secretion by HaCaT cells was quite low and independent of mechanical stretch, suggesting that its effect on cell migration might be ignored. By contrast, EGF secreted by HF cells could play a pivotal role in regulating cell migration since it yielded higher contents as compared to those for HaCaT cells and was able to be further up-regulated by mechanical stretch.

### Asymmetry of HaCaT migration is abolished by chelating EGF

**Figure 8:**
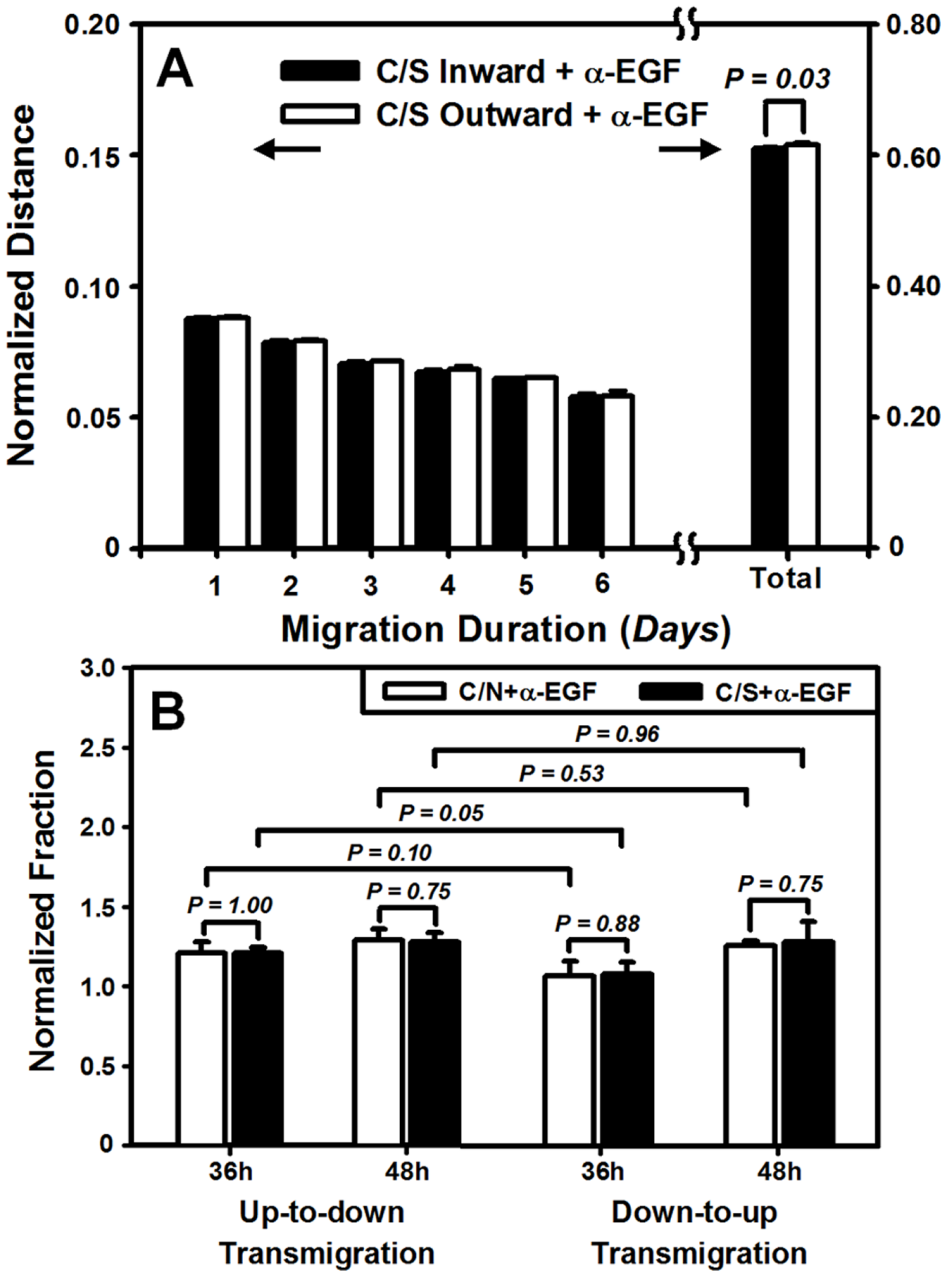
Inhibition of EGF in lateral (*A*) or transmembrane (*B*) migration of HaCaT cells. Data were presented as the mean ± SD of cell migration distance normalized by initial width of HaCaT cells at *t* = 0 day (*A*) or of fraction of transmigrated HaCaT cells at *t* = 36, 48 h normalized to that at *t* = 24 h in respective cases (*B*). Data at *t* = 24 h were not presented for the sake of clarity.

To test the above hypothesis, we conducted the cell migration measurements using blocking mAbs to EGF. For HaCaT lateral migration, the outward *accumulative* distance was significantly reduced from 0.69±0.01 (*cf*. Fig. 2*D*) to 0.62±0.00 (Fig. 8*A*) (*P*<0.01) when anti-EGF Abs were present in the holder box of tensile device, indicating that EGF is a critical cytokine for HaCaT migration. Moreover, the inward or outward migration distance was found to be similar (0.61±0.00 *vs*. 0.62±0.00, *P* = 0.03) (Fig. 8*A*), implying that the asymmetric migration was remarkably inhibited after chelating EGF.

For HaCaT transmigration, blocking EGF in the absence of stretch (*open bars*) slightly enhanced the *up-to-down* migration from 1.12 to 1.21 at *t* = 36 h (*P* = 0.11) or from 1.17 to 1.29 at *t* = 48 h (*P* = 0.14) but dramatically reduced *down-to-up* migration from 1.26 to 1.07 at *t* = 36 h (*P*<0.04) or from 1.49 to 1.26 at *t* = 48 h (*P*<0.01), when comparing the data between EGF-free (Fig. 8*B*) and EGF-presenting (*cf*. Fig. 5*A, B*) cases. Similar tendency was found in the presence of stretch (*solid bars*) where the presence anti-EGF Abs resulted in the enhanced *up-to-down* migration (1.04–1.06 *vs*. 1.21–1.28) but the reduced *down-to-up* migration (1.29–1.49 *vs*. 1.09–1.28) (Figs. 8*B*, 5*D*). In either of the two transmigration manner, the difference in transmigration between C/N and C/S cases was eliminated at each time points (all the values, *P* = 0.75–1.00) (Fig. 8*B*). Importantly, the nature of asymmetric transmigration between *up-to-down* and *down-to-up* migration was no longer observed in the absence or presence of stretch at given time points (all the values, *P*>0.05) (Fig. 8*B*). Taken together, these results indicated that not only EGF is important in regulating the migration magnitude, but it is also critical to preserve the asymmetric feature of HaCaT migration.

### TGF-α or TGF-β1 has little effect on HaCaT asymmetric migration

**Figure 9:**
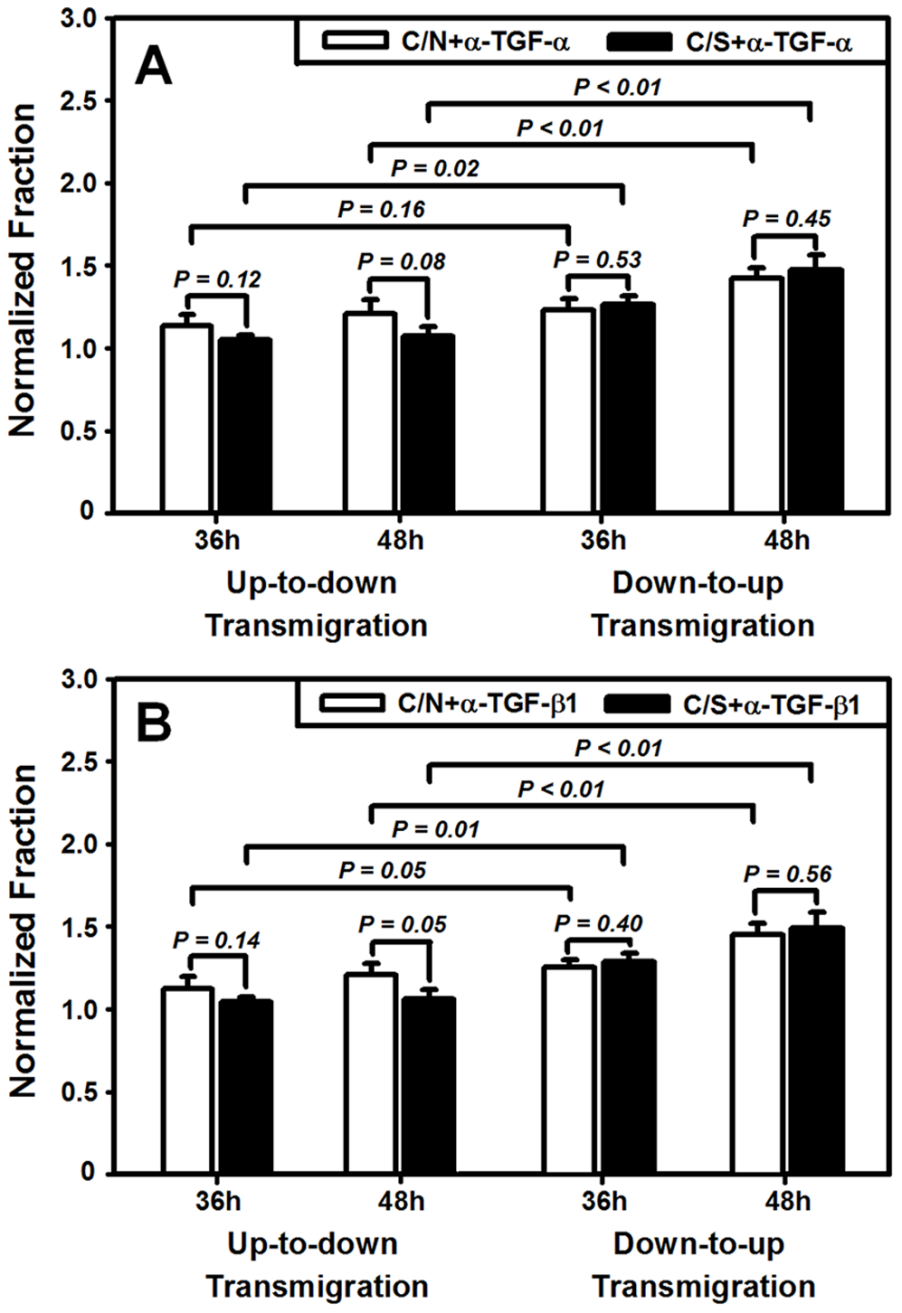
Inhibition of TGF-α (*A*) or TGF-β1 (*B*) in transmigration of HaCaT cells. Data were presented as the mean ± SD of fraction of transmigrated HaCaT cells at *t* = 36, 48 h normalized to that at *t* = 24 h in respective cases. Data at *t* = 24 h were not presented for the sake of clarity.

TGF-α or TGF-β1 functions differently in regulating HaCaT transmigration. To check if other cytokines serve as the potential regulators on asymmetric migration of HaCaT cells, we also conducted the measurements, using anti-TGF-α or TGF-β1 Abs, in 3D assay. Cell transmigration was indifferent in the presence of anti-TGF-α (Fig. 9*A*) or anti-TGF-β1 (Fig. 9*B*) Abs when comparing the data with those in the absence of the antibodies (Figs. 5*C, D*) (all the values, *P* = 0.56–0.98) or between C/N and C/S (all the values, *P* = 0.05–0.56). Asymmetric feature of predominant *down-to-up* migration was reserved almost in all the cases except of the two cases for the C/N transmigration at *t* = 36 h when anti-TGF-α (*P* = 0.16) or anti-TGF-β1 (*P* = 0.05) Abs were present (Figs. 9*A, B*). These results indicated that another member of EGF family, TGF-α, and TGF-β1, played much less roles in regulating the migration magnitude and retaining the asymmetry of HaCaT transmigration.

## Discussion

In dynamic wound healing, keratinocytes migrate laterally along or perpendicularly across the dermis and epidermis under mechanical tension and chemical attractants. The goal of the current work is to elucidate the potential effects and the underlying mechanisms of mechanical stimuli on keratinocyte migration in the presence or absence of fibroblasts in a wound repair model. Herein, we developed a tensile device for testing the lateral migration of HaCaT cells stretched uni-axially on the elastic membrane, modified the transwell assay for quantifying the bi-directional transmigration of HaCaT cells, and combined the both assays for determining the capacity and dynamics of keratinocyte migration regulated by mechanical stretch and cocultured fibroblasts. The novelty of this work mostly lies in that HaCaT cells tend to migrate asymmetrically under mechanical stretch and in the presence of HF cells, in the two sets of tests on lateral (Fig. 1) and transmembrane migration (Fig. 4). Our results also indicated that EGF was mainly secreted by HF (Fig. 7), which plays a pivotal role in mediating the directed, asymmetric migration of HaCaT cells (Figs. 8, 9). Static stretch was able to enhance the EGF secretion by HF cells, which clarifies the direct role of mechanical stretch in HaCaT asymmetric migration. To our knowledge, this is the first time to reveal the asymmetric migration of keratinocytes induced in a cooperative manner of biomechanical and biochemical factors.

Directed cell migration is biologically relevant in many processes such as embryonic development, tissue regeneration after injury, and metastasis of transformed cells. For example, the asymmetric migration of embryonic stem cells is critical for cell rearrangements, which is also a key step for organogenesis [35]. Migration is especially important for germ cells that travel long distances in the embryo to reach their target sites and to form bones and cartilages as well as neurons and glia in the peripheral nervous system [36]. Especially, the asymmetric migration of HaCaT cells is physiologically important in cutaneous genesis and healing. Our data are consistent with the fact that keratinocytes at the epidermis are able to migrate asymmetrically apart from the dermis in 3D microenvironment, as seen in their dominant growth on the top of collagen gel containing human dermal fibroblasts [37]. Moreover, those cells presenting at the wound surface intend to migrate predominantly towards to the scar in 2D environment [38]. In fact, wound healing requires the cooperative interactions between different types of cells, in which keratinocytes in epidermis and fibroblasts in dermis are the prevalent cell types. Their interplay is deterministic to form the functional and aesthetically satisfactory scar. Specifically, the presentation of ECM secreted by fibroblasts is required to initiate the distinct movements of the cells (Figs. 1, 4 and Fig. *S*4). The asymmetric migration of keratinocytes reported here is the result of multiple interactions in the presence of fibroblasts. On the other hand, fibroblast migration could also be regulated by the presence of keratinocytes. Our data collected from both 2D stretch and 3D transwell assays indicated that not only no asymmetric migration is found for HF cells (Figs. 3, 6), but EGF secretion from monocultured HF cells also reserves the same as that for cocultured HF cells (Fig. 7). This adds the information that the feature of fibroblast migration is distinct from that for keratinocytes even when both types of cells are in the same microenvironment. Moreover, fibroblasts play an active role in wound healing by producing many kinds of cytokines. Although keratinocytes are able to stimulate fibroblasts to synthesize growth factors, which further activate the biological functions of keratinocytes in a bi-directional paracrine manner [32], EGF secretion seems not associated with HaCaT presence in the current study, implying the diversity of interplay between fibroblasts and keratinocytes.

Physiological tension of the skin is also critical for inducing the asymmetric migration of keratinocytes. While a large number of investigations have been focused on the effect of mechanical loading on keratinocyte proliferation and the corresponding molecular mechanism, mechanical effect on keratinocyte migration is poorly understood probably due to the technical difficulties. In the current work, we developed a simple, effective tensile device to mimic the skin tension where the uniform uni-axial stretch was obtained and HF cells were placed on one side to HaCaT cells. By applying the device alone or combining it with a modified transwell assay, the lateral or transmembrane migration of HaCaT or HF cells was well determined. Not only our approach is able to test in vitro the keratinocyte or fibroblast migration dynamics under mechanical stretch and in the presence of fibroblasts or keratinocytes, but it also provides clues for explaining why the persistent or intermittent pressure used in VAC technique is able to improve the healing rate and to speed up the wound healing on various types and location of injury [30]. It was also noticed that the static strain used here was 20%, which is well consistent with the average tissue strain of 5–20% used in VAC device predicted using a mathematical model [39]. Our data indicated that HaCaT asymmetric migration is mainly attributed to the coculture with one-side distributed HF cells and to be fostered by static stretch. More physiologically relevant tension, *i.e.*, bi-axial, strain-dependent, or cyclic stretch, will be tested in the future.

It has been documented that the presence of fibroblasts is critical in regulating the secretion of chemotatic growth factors and providing provisional ECM for keratinocyte migration during re-epithelialization [1]. Keratinocytes are not able to migrate efficiently over viable or dead reticular dermis but effectively proliferate, migrate, and differentiate on the reticular dermis by conditioning the surface with live cultured fibroblasts [40], [41]. Among those growth factors and cytokines secreted by fibroblasts, EGF, TGF-α, and TGF-β are assumed to play a role in keratinocyte migration [7], [42]–[44]. In the current work, the asymmetric migration of keratinocytes was evidently inhibited by EGF but not TGF-α and TGF-β1, suggesting that the paracrine pathway of EGF secreted by HF cells has significant impacts on the directed keratinocyte migration and configuration in tissue development and injury repair [45], [46]. Thus, it is reasonably speculated that HaCaT cells simply tends to move away from HF cells, which is mainly due to EGF secreted by HF cells to create an EGF gradient, hence the directionality of HaCaT migration. These results imply a typical case of EGF chemotaxis or possibly a differential migration speed due to different EGF concentrations, which is also consistent with keratinocyte responses to EGF concentrations and gradient in the literatures [47]–[49]. Further studies demonstrated that mechanical stretch subjected to fibroblasts fostered keratinocyte asymmetric migration by increasing EGF secretion (Figs. 7, 8, 9). Our data clearly indicated that EGF is most likely secreted by HF cells but quite less by HaCaT cells, and mainly up-regulated by mechanical stretch but not coculture with HaCaT cells. It is also worth noting that, at the initial phase of stretch (*t* = 1–2 day), EGF level from HF cells was significantly higher than that without stretch and reached the comparable values in between at the late phase (*t* = 3–5 day). These results are consistent with the fact that the initial stretching of the membrane only applies elongational motion to the adhered cells at the initial phase of stretch. Since the membrane will stay unperturbed after initial stretch and then the cells will sense the membrane as stationary substrate at the late phase, the capacity of EGF secretion will be maintained at a constant level. Thus, our data confirmed that the presence of fibroblasts affects the dynamics of keratinocyte migration in a paracrine manner, and EGF is the key growth factor in regulating the asymmetric migration of keratinocytes under mechanical stretch.

While mechanically-induced migration of cocultured HaCaT cells mimics in vitro their responses in physiologically 3D microenvironment, their sensitivity and capacity to undergo cell migration are highly improved in the presence of HF cells and under mechanical stretch. The mechanism underlying this difference involves their differential ability to migrate outward/inward or *up-to-down*/*down-to-up*. Most importantly, the ability of HaCaT migration is asymmetric, which is mainly governed by EGF factor. These results imply the distinct dynamics of keratinocyte migration, especially when the cells are used for cutaneous histogenesis or repairing. It should also be pointed out that, however, HaCaT cell migration could be symmetric if HF cells were distributed uniformly on either side, even though this case is beyond the scope of the current work.

## Conclusions

While mechanically-induced migration of cocultured HaCaT cells mimics *in vitro* their responses in a 3D microenvironment, their sensitivity and capacity to undergo cell migration are highly improved in the presence of HF cells and under mechanical stretch. The mechanism underlying this difference involves their differential ability to migrate outward/inward or *up-to-down*/*down-to-up*. Most importantly, the ability of HaCaT migration is asymmetric and prefers to migrate outwards or *down-to-up*, which is mainly governed by EGF factor mainly secreted by HF cells under mechanical stretch. These results imply the distinct dynamics of keratinocyte migration, especially when the cells are used for cutaneous histogenesis or repairing.

## Supporting Information

Figure S1 **Migration dynamics and accumulative distance of HaCaT monocultured (** ***A*** **,** ***C*** **) or cocultured (** ***B*** **,** ***D*** **) with HF cells on oxygenized membrane in the absence (** ***A*** **,** ***B*** **) or presence (** ***C*** **,** ***D*** **) of mechanical stretch.** Data were presented in the same way as in Figs. 2, 3.
(TIF)

Figure S2 **Migration dynamics and accumulative distance of cocultured HaCaT cells pre-treated by mitomycin C in the absence (** ***A*** **) or presence (** ***B*** **) of mechanical stretch.** Data were collected from at least triplets and presented in the same way as in Figs. 2, 3.
(TIF)

Figure S3 **Migration dynamics and accumulative distance of HF monocultured (** ***A*** **,** ***C*** **) or cocultured (** ***B*** **,** ***D*** **) with HaCaT cells on oxygenized membrane in the absence (** ***A*** **,** ***B*** **) or presence (** ***C*** **,** ***D*** **) of mechanical stretch.** Data were presented in the same way as in Figs. 2, 3.
(TIF)

Figure S4 **Illustration of integrating the mechanical stretch assay and the modified transwell assay for monocultured or cocultured HaCaT cells with HF cells.** 10^5^/ml HaCaT cells were seeded on apical or basolateral side of transwell filter with 8-µm diameter pores pre-coated with collagen I on basolateral or apical side. HF cells were seeded on PDMS membrane mounted to the tensile device, and then cocultured with HaCaT cells presented onto apical or basolateral side. In the growth factors blocking tests, neutralizing Abs were added into the holder box of tensile device or the lower compartment of transwell filter.
(TIF)

Figure S5 **Transmigration of HaCaT cells in the presence or absence of EGF.** Staining of HaCaT cells having transmigrated across the transwell filter in the *up-to-down* (*A*) or *down-to-up* (*B*) manner by crystal viole. EGF-presenting (*left column*) and EGF-free (*right column*) cases in each panel were compared for non-stretched, monocultured (1^st^ *row*) or cocultured (2^nd^ *row*) as well as stretched, cocultured (3^rd^ *row*) HaCaT cells.
(TIF)

Movie S1 **Time course of HaCaT cells moving leftward on collagen I-coated PDMS membrane from day 0 to day 6.**
(MP4)

Movie S2 **Time course of HaCaT cells moving rightward on collagen I-coated PDMS membrane from day 0 to day 6.**
(MP4)
