# Supplementary figures and images for: Asymmetric Migration of Human Keratinocytes under Mechanical Stretch and Cocultured Fibroblasts in a Wound Repair Model
Source: PLoS One. 2013 Sep 23;8(9):e74563. doi: 10.1371/journal.pone.0074563 (PMC3781156; doi:10.1371/journal.pone.0074563)

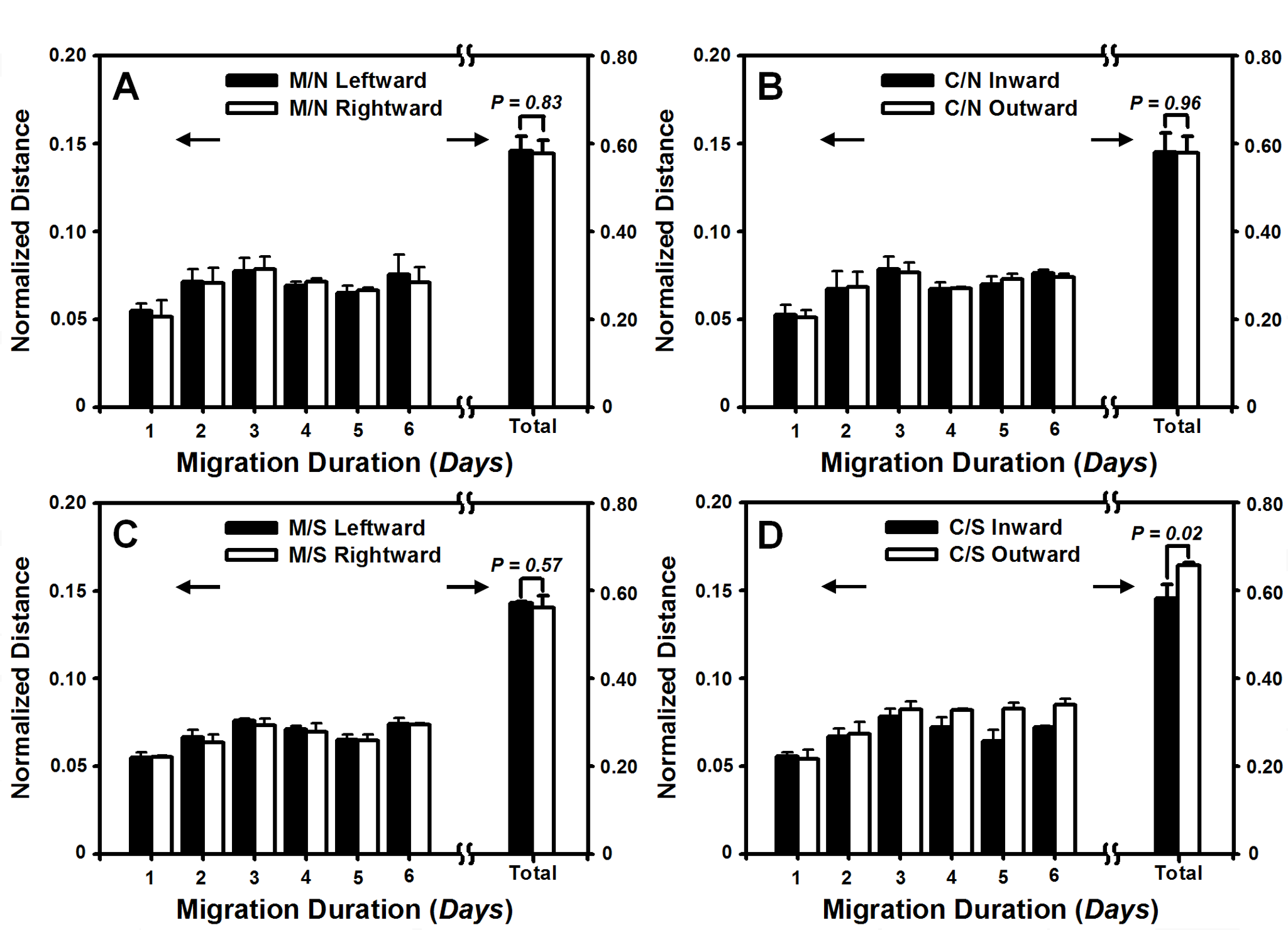

Supplement: Figure S1 — Migration dynamics and accumulative distance of HaCaT monocultured ( A , C ) or cocultured ( B , D ) with HF cells on oxygenized membrane in the absence ( A , B ) or presence ( C , D ) of mechanical stretch. Data were presented in the same way as in Figs. 2, 3. (TIF) [file pone.0074563.s001.tif]

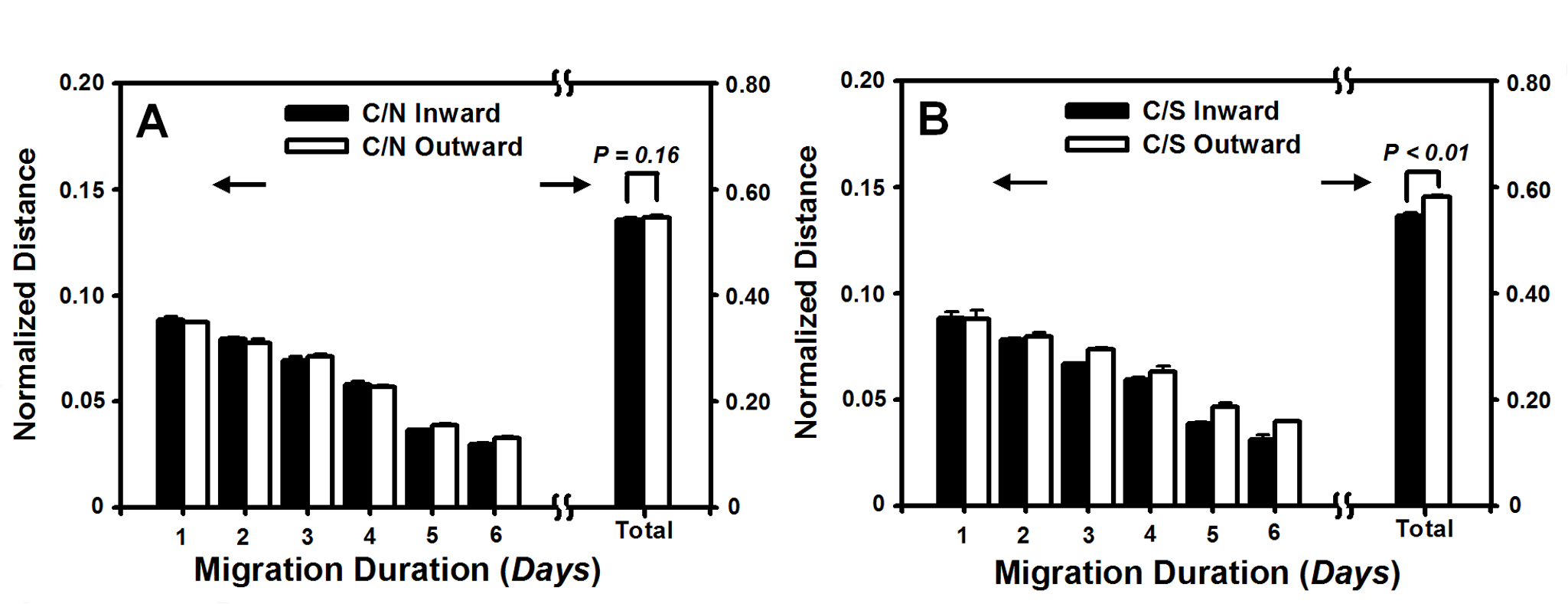

Supplement: Figure S2 — Migration dynamics and accumulative distance of cocultured HaCaT cells pre-treated by mitomycin C in the absence ( A ) or presence ( B ) of mechanical stretch. Data were collected from at least triplets and presented in the same way as in Figs. 2, 3. (TIF) [file pone.0074563.s002.tif]

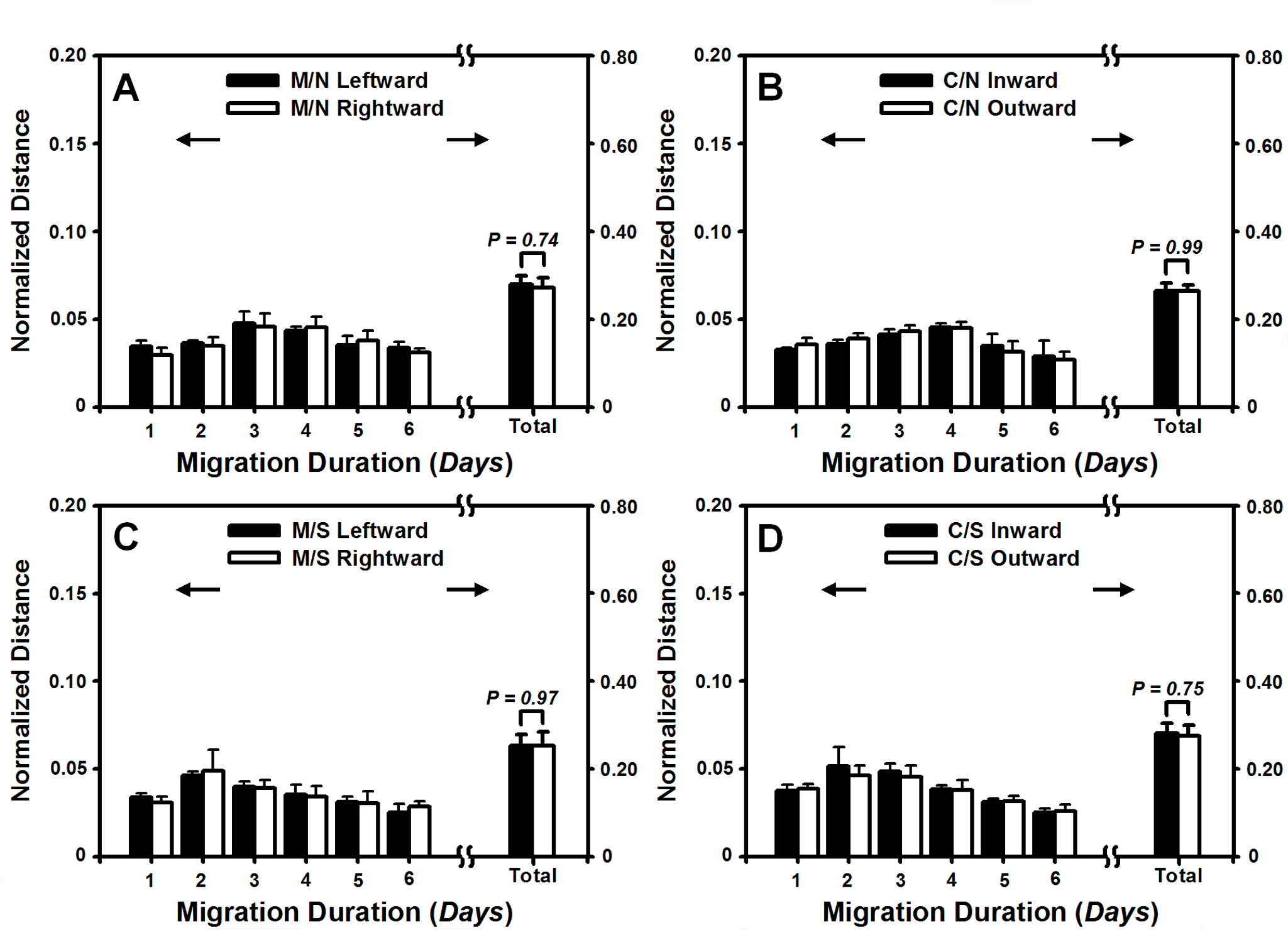

Supplement: Figure S3 — Migration dynamics and accumulative distance of HF monocultured ( A , C ) or cocultured ( B , D ) with HaCaT cells on oxygenized membrane in the absence ( A , B ) or presence ( C , D ) of mechanical stretch. Data were presented in the same way as in Figs. 2, 3. (TIF) [file pone.0074563.s003.tif]

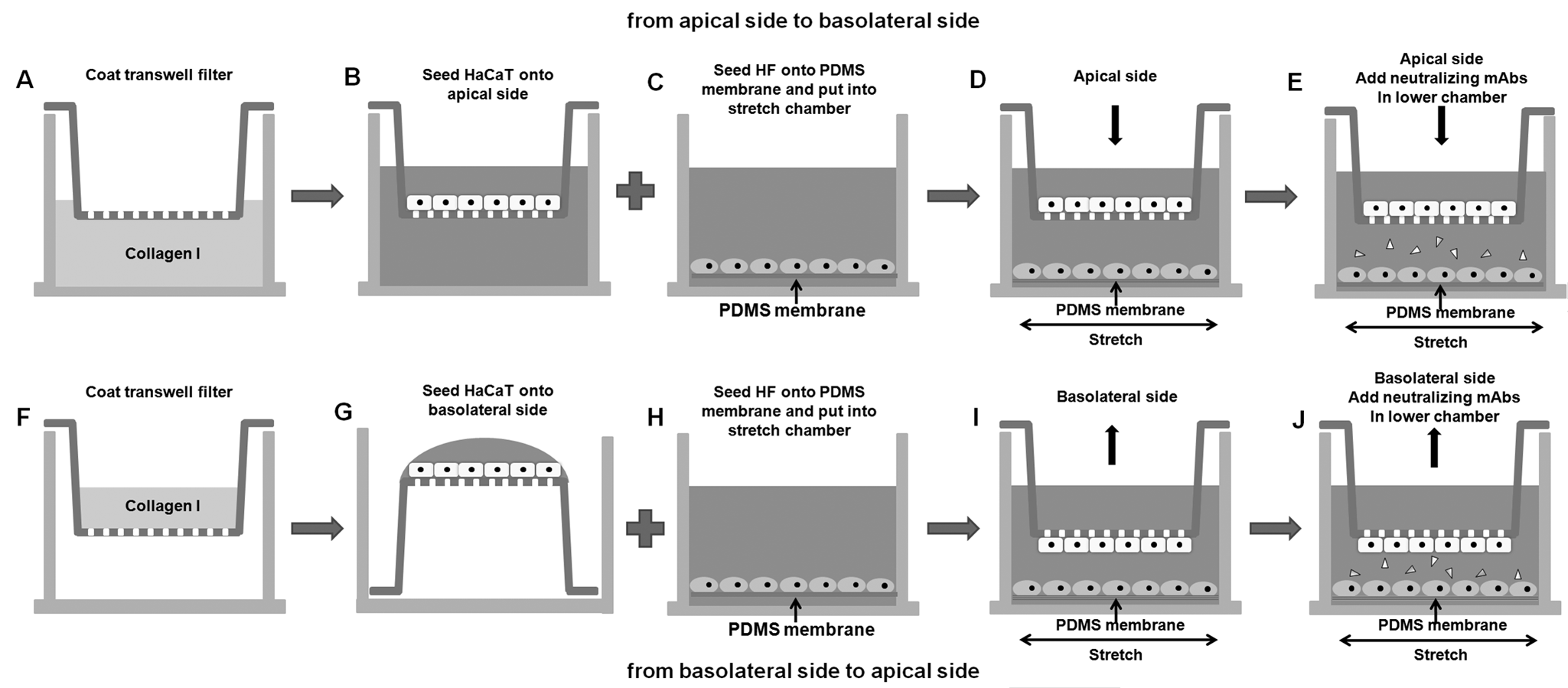

Supplement: Figure S4 — Illustration of integrating the mechanical stretch assay and the modified transwell assay for monocultured or cocultured HaCaT cells with HF cells. 105/ml HaCaT cells were seeded on apical or basolateral side of transwell filter with 8-µm diameter pores pre-coated with collagen I on basolateral or apical side. HF cells were seeded on PDMS membrane mounted to the tensile device, and then cocultured with HaCaT cells presented onto apical or basolateral side. In the growth factors blocking tests, neutralizing Abs were added into the holder box of tensile device or the lower compartment of transwell filter. (TIF) [file pone.0074563.s004.tif]

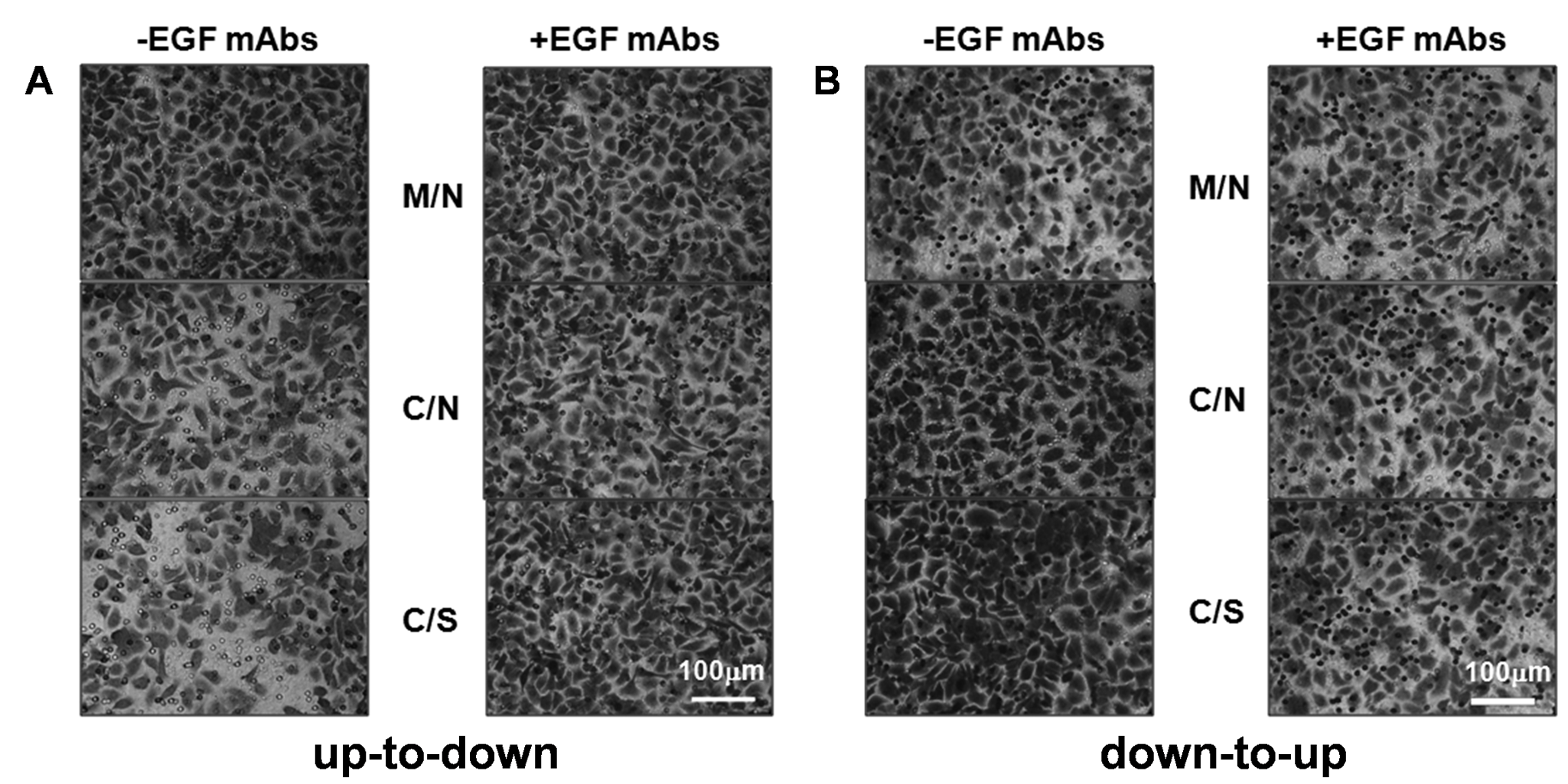

Supplement: Figure S5 — Transmigration of HaCaT cells in the presence or absence of EGF. Staining of HaCaT cells having transmigrated across the transwell filter in the up-to-down (A) or down-to-up (B) manner by crystal viole. EGF-presenting (left column) and EGF-free (right column) cases in each panel were compared for non-stretched, monocultured (1st row) or cocultured (2nd row) as well as stretched, cocultured (3rd row) HaCaT cells. (TIF) [file pone.0074563.s005.tif]
